# Two AOS genes attributed to familial exudative vitreoretinopathy with microcephaly: Two case reports

**DOI:** 10.1097/MD.0000000000024633

**Published:** 2021-03-05

**Authors:** Zhiyan Tao, Shaochong Bu, Fang Lu

**Affiliations:** a Department of Ophthalmology, West China Hospital, Sichuan University, Chengdu, Sichuan Province; b Tianjin Medical University Eye Hospital and Eye Institute, Tianjin, China.

**Keywords:** Adams-Oliver syndrome, familial exudative vitreoretinopathy, microcephaly, whole exon sequencing

## Abstract

**Rationale:**

Familial exudative vitreoretinopathy (FEVR) is an inherited disorder, which is mostly reported to be associated with the mutation of genes involved in the Wnt signaling pathway related to β-catenin. To the best of our knowledge, the involvement of Adams-Oliver syndrome (AOS) genes in FEVR patients have not been reported before.

**Patient concerns:**

Two patients with FEVR presented with microcephaly. One of them showed slight scarring of the scalp vertex which is a typical manifestation of AOS. The whole exon sequencing confirmed the diagnosis of AOS with 2 AOS-gene mutations at *DOCK6* and *ARHGAP31*. Further clinical examination revealed that their parents with the same mutations showed FEVR-like vascular anomalies.

**Diagnosis:**

Both patients were diagnosed with AOS through whole exon sequencing, and they presented with some FEVR-like retinopathy including retinal detachment.

**Interventions:**

Both patients received vitrectomy for tractional retinal detachment with proliferative vitreoretinopathy. During the follow-up, 1 patient received additional laser photocoagulation for tractional retinal detachment.

**Outcomes:**

The 2 patients remained stable in the latest follow up after the treatment.

**Lessons:**

Microcephaly could be associated with some form of retinopathy. We proposed that mutation of *DOCK6* and *ARHGAP31* genes could be the possible cause of FEVR associated with microcephaly. Our study suggested that these genes may be candidate genes of FEVR.

## 1 Introduction

Familial exudative vitreoretinopathy (FEVR) is an inherited disorder in which the clinical appearance varies considerably among all affected patients.^[1]^ Patients affected showed diverse manifestations, from only avascularity in the peripheral retina detected under fundus fluorescein angiography (FFA) to extensive retinal detachment. The peripheral angiogenesis defect results in retina hypoxia, which is the underlying cause of the hyperpermeable retinal blood vessels, neovascularization, fibrotic vitreoretinal traction, retinal folds, and retinal detachments. In severely affected patients, the condition could result in irreversible blindness.^[2]^

The genes involved in the Wnt signaling pathway have been identified to be closely associated with FEVR, such as *FZD4* gene (604579), *NDP* gene (300658), *LRP5* gene (603506), *TSPAN12* gene (613138), *ZNF408* gene (616454), and *KIF11* (148760). The current report suggested that 2 genes of Adams-Oliver syndrome (AOS) (OMIM 100300) could be associated with FEVR.

## 2 Case report

This study was approved by the ethics committee of West China Hospital, Sichuan University. The guardians of patients have provided informed consents for the publication of the cases. The 2 patients were preliminarily diagnosed with FEVR by experienced retinal specialist based on the typical ocular manifestations. The diagnosis of microcephaly was made based on the occipitofrontal circumference (OFC) information. The blood samples were collected from the probands and family members for mutation screening by whole exon sequencing (WES) at a third-party company. The average sequencing depth of WES was 200, and the average coverage of the target regions was 99.75%. The fraction of targets covered with at least 10X was 99.30% and that with 20X was 98.17%. Whole-exome capture was carried out using the Nimblegen Human All Exon Kit (Roche, Basel, Switzerland) and high-throughput sequencing by the Illumina HiSeq sequencer (Illumina, San Diego, CA). The biological function prediction for the proteins was performed by using prediction software Provean, SIFT, Polyphen2_HDIV, Polyphen2_HVAR, mutationtaster, M-CAP, and REVEL. The pathogenicity of each variant was classified into “pathogenic,” “likely pathogenic,” “uncertain significance.” Sanger sequencing was performed to validate the pathogenic variants identified by WES. The variant sequences were amplified with an annealing temperature predicted by software DNAclub. Polymerase Chain Reaction (PCR) products were sequenced with ABI 3730XL (Thermo Fisher Scientific Inc, Waltham, MA), followed by analysis with the DNASTAR 5.0 software (DNASTAR, Inc, Madison, WI).

## 3 Case 1

**Figure 1:**
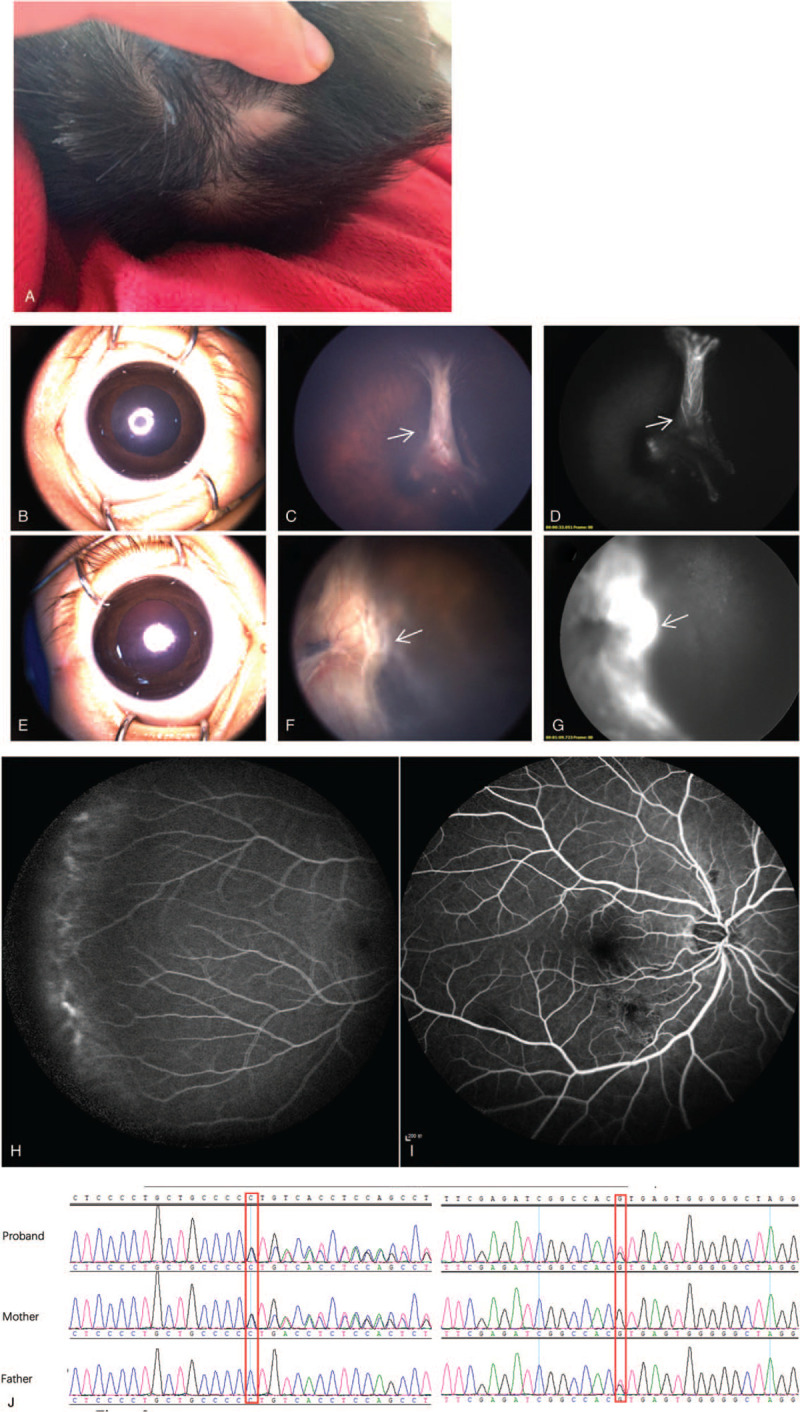
Family's fundus and FFA pictures of case 1: A showed the scarring of the scalp vertex of proband 2; B to D showed the right eye character of the patient; B showed the anterior segment, C showed the fundus and D was FFA picture. E to G presented the corresponding elements of the left eye. C and D indicated the vascularized mass in the right eye. F and G exhibited the vascularized mass in the left eye (white arrow). H presented the FFA picture of the mother and I for the father. J Sanger sequencing showed the mutations, c.3190_3191del and c.4491+1G>T were originated from the maternal and paternal lineage respectively. FFA = fundus fluorescein angiography.

Proband 1 was a 4-month-old male with OFC of 35.1 cm at 2 months old which was 3 standard deviations beneath the standard. Physical examinations showed slight scarring of the scalp vertex (Fig. 1A) and no obvious anomaly in the anterior chamber (Fig. 1B, E). The fundus examination showed a vascularized tractional band in front of the optic disc in the right eye (Fig. 1C, D) and traction retinal detachment with anterior extension in the left eye (Fig. 1F, G). In addition, the patient showed developmental retardation mentally. Both parents showed slight retinal vascular anomalies on FFA examination (Fig. 1H, I).

**Figure 2:**
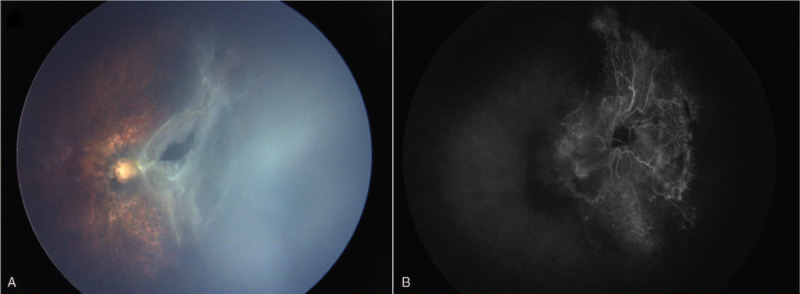
The fundus picture and fundus fluorescein angiography results of the right eye after the vitrectomy. The result showed that the retina reattached with chaotic retinal vessels.

The right eye received vitrectomy for tractional retinal detachment associated with proliferative vitreoretinopathy. The retina reattached after the surgery (Fig. 2A) with chaotic retinal vessels without leakage on FFA (Fig. 2B). The patient has been stable with follow-up every 3 months.

WES revealed a pathogenic mutation at *DOCK6* of AOS (OMIM 100300). The proband carried a matrilineal frameshift pathogenic variant, c.3190_3191del, and a patrilineal splicing pathogenic variant, c.4491+1G>T in *DOCK6* (Fig. 1J). Based on the results of WES and clinical manifestations, the patient was diagnosed with AOS associated with FEVR.

## 4 Case 2

**Figure 3:**
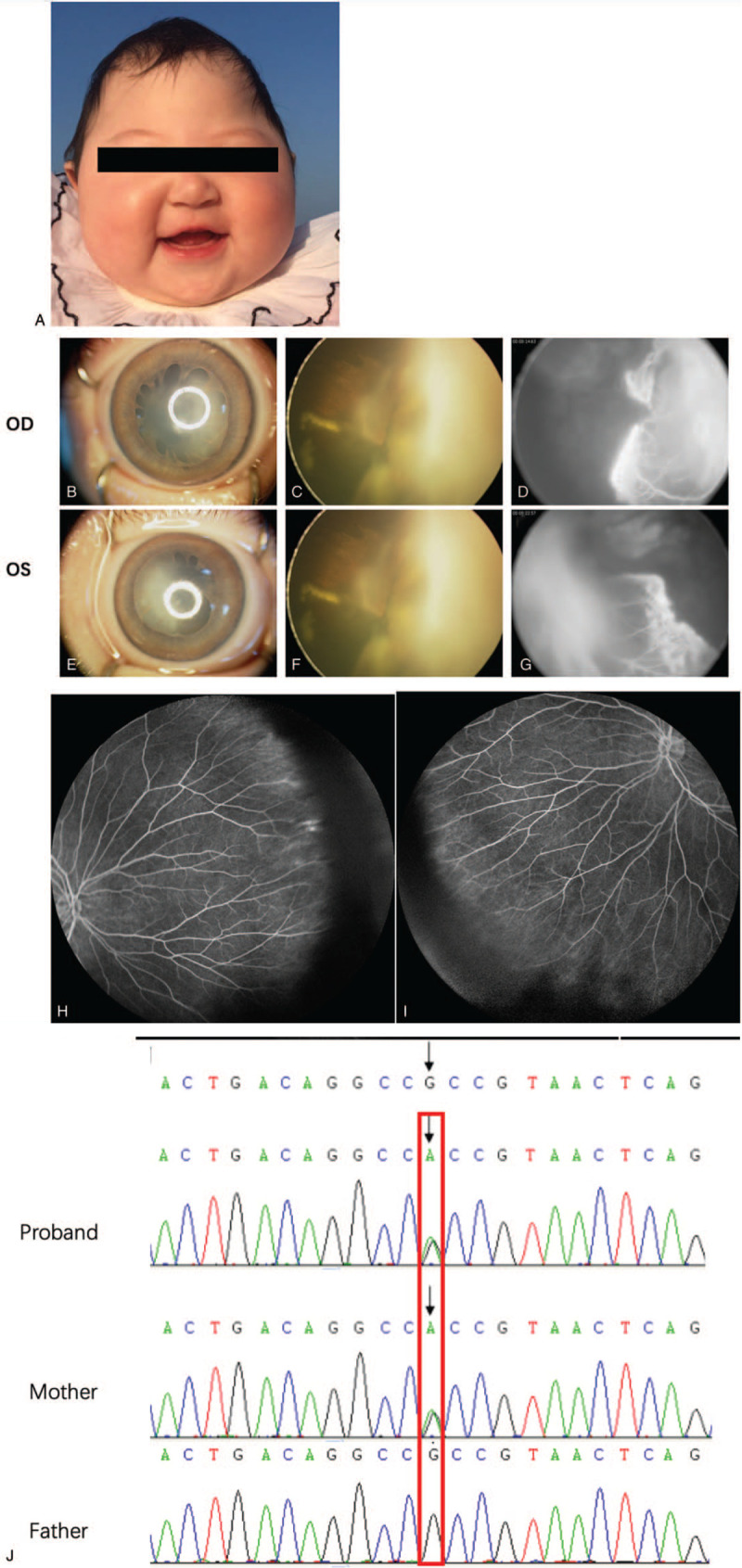
Family's fundus and FFA pictures of case 2: A showed the microcephaly appearance of proband 1; B to D showed the right eye character of the patient; B showed anterior segment, C presented fundus and D was FFA picture. E to G presented the corresponding elements of the left eye. C and F indicated the tractional and exudative retinal detachment with massive hard exudates. D and G exhibited new pathologic vessels with heavy leakage of fluorescein. H and I presented the right eye and left eye of the mother, respectively. J Sanger sequencing showed the mutation, c.3524G>A was inherited from the maternal lineage in which the red box marked the mutation loci. FFA = fundus fluorescein angiography.

Proband 2 was a 4-month-old female with OFC of 37.5 cm at 3 months old which was 2 standard deviations beneath the standard (Fig. 3A). Physical examinations showed bilateral microphthalmia, persistent pupillary membrane, and lens opacity (Fig. 3B, E). Retinal examination revealed proliferative vitreoretinopathy and retinal detachment with massive exudates in the posterior pole (Fig. 3C, F). FFA showed extremely chaotic retinal vessels originated from the optic disc with intensive leakage on FFA (Fig. 3D, G). Her mother was diagnosed with FEVR based on the peripheral retinal vascular anomalies on FFA (Fig. 3H, I).

**Figure 4:**
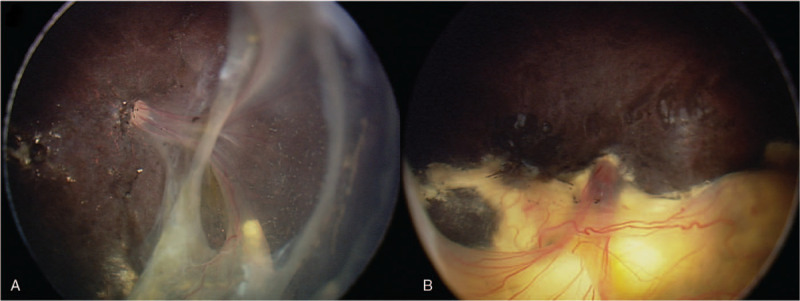
Fundus pictures of proband 2 fourteen months after the treatment. The fundus photos showed the retinae in both eyes remained attached and stable.

The patient received bilateral vitrectomy and lensectomy, and additional laser photocoagulation on the left eye during follow-up. Since then she has been followed up at 3 months interval for 2 years. The exudate in the right eye was absorbed completely (Fig. 4A) and those in the left eye remained as a solid mass (Fig. 4B).

The WES showed a pathogenic mutation of *ARHGAP31*. A missense pathogenic variant of AOS, c.3524G>A of *ARHGAP31* was found in proband 2 and her mother (Fig. 3J). Based on the results of WES and clinical manifestations, the patient was diagnosed with AOS associated with FEVR.

## 5 Discussion

Microcephaly and chorioretinopathy are presented in several diseases for the genetic heterogeneity. It has also been established that the genes involved in microcephaly with FEVR were the cause for varied diseases. A recent study on patients from 10 families of FEVR with microcephaly reported that 2 LRP5 mutations can cause FEVR with microcephaly,^[3]^ while AOS-related genes had not been linked to FEVR with microcephaly.

AOS is a rare developmental disorder, typically appeared as the aplasia cutis congenita of the scalp vertex and terminal transverse limb defects, while the vascular anomalies such as retinal hypervascularization has been occasionally observed.^[4]^ AOS cases reported in China usually presented primarily as epilepsy but few of retinopathy. Although the 2 cases in our study manifested atypical AOS, the genetic evidence supported the diagnosis of AOS. We have analyzed the family history of the 2 AOS probands to verify the involvement of the 2 genes on FEVR. For proband 1, one mutation inherited from the maternal and the other from the paternal lineages, both parents showed mild vascular retinopathy (Fig. 1H, I). For proband 2, the mutation originated from the maternal lineage who was also diagnosed with FEVR (Fig. 3H, I). The pedigrees indicated a hypothesis that these 2 AOS-related genes may be the candidate genes of FEVR, showing incomplete penetrance in the patients and their parents.

To test our hypothesis, we searched and analyzed the clinical manifestations and functions of the aforementioned genes. *ARHGAP31* and *DOCK6* are known to be responsible for AOS.^[5,6]^*DOCK6* was reported to be associated with AOS2 for abnormal actin cytoskeleton organization,^[5]^ and the main function of *ARHGAP31* was reported to be associated with AOS1 including syndromic cutis aplasia and limb anomalies.^[6]^ As for AOS, although the typical manifestations of AOS do not appear in the eye, some AOS patients with FEVR-like retinopathy were reported previously, such as the extensive fibrovascular resulting in traction retinal detachment,^[7,8]^ peripheral avascular retina and microphthalmia with microcornea,^[9]^ and radial retinal folds involving the macula.^[10]^ In our report, the AOS patients had severe FEVR, which was classified as phase 4 or phase 5 with the retinal detachment. Their main pathology was presented in eyes, which were consistent with the features of Norrie disease.

*ARHGAP31* encodes Rho GTPase-activating protein 31, which acts as a negative regulator for Rho GTPases Rac1 and Cdc42. The GTPase-activating protein 31 constitutively interacts with vascular endothelial growth factor receptor 2 (VEGFR2) and is required for the vascular endothelial growth factor (VEGF)-mediated angiogenesis, VEGF-induced endothelial cell migration, and capillary formation.^[11]^ The mutation in *ARHGAP31* was reported to impede the embryonic vascular development and VEGF-induced signaling.^[11]^*DOCK6* is a member of DOCK180-related proteins, which are atypical types of guanine–nucleotide exchange factors for Rac and/or Cdc42, playing key roles in essential cellular functions such as regulations of the cytoskeletal organization, phagocytosis, cell migration, polarity formation, and differentiation.^[12]^ Meanwhile, all pathogenic genes of FEVR, namely, *FZD4*, *LRP5*, and *NDP*, are involved in the Wnt signaling and related to β-catenin. It has been proven that the nuclear accumulation of β-catenin in response to Wnt signaling requires Rac1 activation. The genetic ablation of Rac1 in the limb bud ectoderm of mouse embryos would disrupt the canonical Wnt signaling.^[13]^ Lutze et al have also described noncanonical Wnt-signaling for the differentiation of lymphatics and the extension of lymphangiogenesis via Rac and c-Jun N-terminal kinase, suggesting that Rac has effects on the Wnt signaling.^[14]^ Therefore, we proposed that genes *ARHGAP31* and *DOCK6* may interact with Wnt signaling through Rho GTPase Rac1 and Cdc42, which plays important role in the pathogenesis of FEVR.

In conclusion, we reported 2 AOS patients with severe FEVR carried mutations in *DOCK6* and *ARHGAP31*, which indicated that the 2 genes could be candidate genes of FEVR.

## Author contributions

**Conceptualization:** Fang Lu.

**Data curation:** Zhiyan Tao.

**Writing – original draft:** Zhiyan Tao.

**Writing – review & editing:** Shaochong Bu, Fang Lu.
